# Cancer Predisposition Cascade Screening for Hereditary Breast/Ovarian Cancer and Lynch Syndromes in Switzerland: Study Protocol

**DOI:** 10.2196/resprot.8138

**Published:** 2017-09-20

**Authors:** Maria C Katapodi, Valeria Viassolo, Maria Caiata-Zufferey, Christos Nikolaidis, Rosmarie Bührer-Landolt, Nicole Buerki, Rossella Graffeo, Henrik Csaba Horváth, Christian Kurzeder, Manuela Rabaglio, Michael Scharfe, Corinne Urech, Tobias E Erlanger, Nicole Probst-Hensch, Karl Heinimann, Viola Heinzelmann-Schwarz, Olivia Pagani, Pierre O Chappuis

**Affiliations:** ^1^ Nursing Science Faculty of Medicine University of Basel Basel Switzerland; ^2^ University of Michigan School of Nursing Ann Arbor, MI United States; ^3^ Unit of Oncogenetics and Cancer Prevention Division of Oncology Geneva University Hospitals Geneva Switzerland; ^4^ University of Applied Sciences and Arts of Southern Switzerland Manno Switzerland; ^5^ University Clinic for Medical Oncology Inselspital Bern Bern Switzerland; ^6^ Women’s Clinic and Gynecological Oncology University Hospital Basel University of Basel Basel Switzerland; ^7^ Institute of Oncology (IOSI) and Breast Unit (CSSI) of Southern Switzerland Bellinzona Switzerland; ^8^ University Clinic for Visceral Surgery and Medicine Inselspital Bern Bern Switzerland; ^9^ Clinical Trials Unit Department of Clinical Research, University Hospital Basel University of Basel Basel Switzerland; ^10^ Swiss Tropical and Public Health Institute University of Basel Basel Switzerland; ^11^ Medical Genetics University Hospital Basel University of Basel Basel Switzerland; ^12^ Division of Genetic Medicine Geneva University Hospitals University of Geneva Geneva Switzerland

**Keywords:** public health genetics, public health interventions, family communication, cancer surveillance, patient-provider communication, quality of life, psychosocial support, family-based interventions

## Abstract

**Background:**

Breast, colorectal, ovarian, and endometrial cancers constitute approximately 30% of newly diagnosed cancer cases in Switzerland, affecting more than 12,000 individuals annually. Hundreds of these patients are likely to carry germline pathogenic variants associated with hereditary breast ovarian cancer (HBOC) or Lynch syndrome (LS). Genetic services (counseling and testing) for hereditary susceptibility to cancer can prevent many cancer diagnoses and deaths through early identification and risk management.

**Objective:**

Cascade screening is the systematic identification and testing of relatives of a known mutation carrier. It determines whether asymptomatic relatives also carry the known variant, needing management options to reduce future harmful outcomes. Specific aims of the CASCADE study are to (1) survey index cases with HBOC or LS from clinic-based genetic testing records and determine their current cancer status and surveillance practices, needs for coordination of medical care, psychosocial needs, patient-provider and patient-family communication, quality of life, and willingness to serve as advocates for cancer genetic services to blood relatives, (2) survey first- and second-degree relatives and first-cousins identified from pedigrees or family history records of HBOC and LS index cases and determine their current cancer and mutation status, cancer surveillance practices, needs for coordination of medical care, barriers and facilitators to using cancer genetic services, psychosocial needs, patient-provider and patient-family communication, quality of life, and willingness to participate in a study designed to increase use of cancer genetic services, and (3) explore the influence of patient-provider communication about genetic cancer risk on patient-family communication and the acceptability of a family-based communication, coping, and decision support intervention with focus group(s) of mutation carriers and relatives.

**Methods:**

CASCADE is a longitudinal study using surveys (online or paper/pencil) and focus groups, designed to elicit factors that enhance cascade genetic testing for HBOC and LS in Switzerland. Repeated observations are the optimal way for assessing these outcomes. Focus groups will examine barriers in patient-provider and patient-family communication, and the acceptability of a family-based communication, coping, and decision-support intervention. The survey will be developed in English, translated into three languages (German, French, and Italian), and back-translated into English, except for scales with validated versions in these languages.

**Results:**

Descriptive analyses will include calculating means, standard deviations, frequencies, and percentages of variables and participant descriptors. Bivariate analyses (Pearson correlations, chi-square test for differences in proportions, and t test for differences in means) will assess associations between demographics and clinical characteristics. Regression analyses will incorporate generalized estimating equations for pairing index cases with their relatives and explore whether predictors are in direct, mediating, or moderating relationship to an outcome. Focus group data will be transcribed verbatim and analyzed for common themes.

**Conclusions:**

Robust evidence from basic science and descriptive population-based studies in Switzerland support the necessity of cascade screening for genetic predisposition to HBOC and LS. CASCADE is designed to address translation of this knowledge into public health interventions.

**Trial Registration:**

ClinicalTrials.gov NCT03124212; https://clinicaltrials.gov/ct2/show/NCT03124212 (Archived by WebCite at http://www.webcitation.org/6tKZnNDBt)

## Introduction

Breast, colorectal, ovarian, and endometrial cancers constitute approximately 30% of newly diagnosed cancer cases in Switzerland, affecting more than 12,000 individuals annually [1]. About 2%-15% of incident cases are associated with known hereditary cancer syndromes. Several hundred Swiss patients diagnosed with any of these cancers are likely to carry known pathogenic germline variants [2]. Approximately 5-10% of breast cancer cases and 10%-15% of epithelial ovarian cancer cases develop due to single gene mutations that are passed down in the family, such as the breast cancer 1 (*BRCA1*) and breast cancer 2 (*BRCA2*) genes [3,4]. Germline *BRCA* mutations are associated with most hereditary breast and ovarian cancer (HBOC) cases. Women with *BRCA* mutations have a 55%-70% risk of breast cancer and 17%-59% risk of ovarian cancer by age 70, while the corresponding lifetime risks in the general population are 12% and 1.3%, respectively [5-7]. HBOC cases have an increased risk of cancer at a younger age, often before recommendations for routine screening apply [8,9]. The prevalence of *BRCA* mutations varies considerably among ethnic groups and geographical areas. In Caucasian populations, the prevalence of *BRCA* pathogenic variants is estimated at 1:400 to 1:500, whereas the frequency of three founder mutations in the Ashkenazi Jewish population is 1:40 [10-12]. About 21% of Swiss breast cancer patients are diagnosed younger than 50 years old, which may indicate genetic susceptibility [13,14].

Lynch syndrome (LS), previously known as hereditary nonpolyposis colorectal cancer, is an inherited disorder, associated with 22%-74% lifetime risk for colorectal cancer, 14%-71% risk for endometrial cancer, 3%-22% risk for ovarian cancer, up to 13% risk for gastric cancer, and up to 25% risk for urothelial cancer [15]. LS accounts for about 2%-5% of colorectal cancer and endometrial cancer burden, as well as increased risk for several other malignancies including gastric, ovarian, small bowel, urinary and biliary tract, pancreatic, and sebaceous gland tumors [16]. Individuals with LS have a 10%-74% risk of colorectal cancer, and a 14%-71% risk of endometrial cancer by age 70, while the corresponding rates in the general population are 5.5% and 2.7%, respectively [17,18]. A hallmark of LS is early age of onset, usually before the age of 50 at which recommendations for routine screening apply [15,19]. Most LS-related tumors are characterized by a high level of microsatellite instability (MSI-H), which is distinctive of cancers with a defective DNA mismatch repair (MMR) mechanism [20]. Diagnosis of LS involves a sequential process including prescreening with MSI testing and immunohistochemistry analysis to determine expression of the main MMR proteins (*MLH1, MSH2, MSH6, PMS2*) in tumor tissues. Additional *MLH1* promoter methylation testing eliminates the possibility of loss of *MLH1* expression due to epigenetic mechanisms or identification of a somatic *BRAF* pathogenic variant (c.1799T>A/p.V600E). In the case of pathological prescreening results, germline analyses of two or more MMR genes (*MLH1/PMS2* and/or *MSH2/MSH6*) and search for *EPCAM* deletions confirm the diagnosis. Germline mutations in the *MLH1* and *MSH2* genes account for up to 90% of LS cases, whereas *MSH6* and *PMS2* mutations account for most of the remaining cases [21]. The Amsterdam Criteria II and Revised Bethesda Guidelines are used in clinical practice for identifying individuals concerned about LS [22]. These guidelines are not sensitive enough and may miss up to 30% of LS cases [23]. Even if the population prevalence of LS is estimated at 1:440 [24], LS is vastly underdiagnosed compared to HBOC.

Germline mutations connected to HBOC and LS are inherited in an autosomal dominant manner. De novo mutations are rare in these syndromes. For every identified mutation carrier, there are multiple family members who may carry the same mutation. First- and second-degree relatives and first cousins of known carriers have 50%, 25%, and 12.5% probability for inheriting the respective cancer predisposition. The availability of cancer genetic services (counseling and testing) for HBOC and LS is a significant milestone for effective cancer prevention and control [25]. When a pathogenic variant is identified, relatives can be tested with 100% accuracy [26]. Genetic counseling can educate patients and cancer-free individuals about cancer risk and management options according to mutation status. Physicians’ attitudes [27] and coverage of cost of tests and gene panels by health insurance influence whether genetic testing is performed or not [28].

A Swiss study reported that about 11% of all breast cancer patients and 25% of those with a strong family history used genetic services [29]. These figures are lower for LS-related colorectal and endometrial cancer patients, suggesting that many Swiss mutation carriers and their family members may not benefit from advances in health care technology and medical diagnostics. HBOC and LS patients are at an increased risk of secondary cancers and can benefit from intensive surveillance, pharmacoprevention, or prophylactic surgery. Prophylactic surgery such as mastectomy, bilateral salpingo-oophorectomy, and hysterectomy should be discussed with women affected with HBOC or LS [30]. Subtotal colectomy can be considered for LS patients with colorectal cancer [18]. Family members who test positive benefit from high-risk management care starting at age 25-30, or 10 years before the earliest age of breast cancer onset in the family. This care can include annual breast magnetic resonance imaging, mammograms, pelvic ultrasound for women (HBOC) [31], and annual colonoscopy starting at age 20-25, or 2-5 years before the earliest age of colorectal cancer onset in the family, whichever comes first (LS) [15,18]. Implementing clinical recommendations and providing high-quality surveillance to patients during survivorship requires excellent coordination of health care services provided in high-risk clinics [32-35].

Mutation carriers identified through complete genetic analyses are asked to communicate test results to relatives and encourage them to use genetic services. This process is highly variable from family to family, with less than 40% of high-risk relatives using genetic services, suggesting a lack of effective communication [36,37]. Lack of understanding of genetic information combined with family conflicts most likely inhibits disclosure of test results to relatives [38,39]. In Switzerland, the Federal Act on Human Genetic Testing (HGTA) is the legal regulation that directly applies to the clinical practice of genetic analysis. HGTA states that a physician is not allowed to disclose genetic test results to anyone except the tested individual or their legal representative. Results can be disclosed to family members, spouses, or partners only with the explicit consent of the tested individual. If the tested individual refuses to disclose this information, if they are deceased, have disappeared, or are unable to consent in the absence of an authorized delegate, the physician can seek help from the expert commission on professional confidentiality. The physician may apply to the appropriate cantonal authority to be released from the duty of professional secrecy if protecting the overriding interests of the family members, spouse, or partner requires that they receive this information. Cantonal authorities may also request an opinion from the Expert Commission for Human Genetic Testing [40]. Interventions designed to facilitate patient-provider and patient-family communication can enhance understanding of genetic information and facilitate the disclosure of test results from carriers to relatives and can contribute to more effective management of hereditary cancer. Several such interventions have been developed and tested in the United States [41-51] but should be adapted before they can be implemented in Switzerland, due to cultural and possibly legal differences.

Cascade screening is the sequential process of identifying and testing blood relatives of a known mutation carrier to determine if additional individuals carry the pathogenic variant, and proposing preventive and other clinical management options to reduce morbidity and mortality [52]. Cascade screening also reassures non-carrier relatives and excludes them from intensive surveillance, making it cost-effective and contributing to personalized medicine [53]. The Centers for Disease Control and Prevention, Office for Public Health Genomics issued evidence-based recommendations justifying genetic testing in affected individuals and relatives when there is a known family history of HBOC or other *BRCA*-related cancers, LS-related colorectal cancer, or familial hypercholesterolemia (FH). These are Tier 1 genetic conditions suitable to promoting translation of scientific breakthroughs in genetics to public health [54]. There are currently no systematic efforts to apply cascade screening for Tier 1 genetic conditions among the general population in Europe apart from the Netherlands, which successfully implemented a cascade screening program for FH. The implementation of this pioneering public health program helped identify more than 28,000 asymptomatic cases [55] and provides proof-of-concept that cascade screening can be applied in other settings [56].

Robust evidence from basic science and descriptive population-based studies in Switzerland support the necessity of cascade screening for HBOC and LS [57-67]. However, there are currently no interventions to translate this knowledge into public health. Researchers know little about the cancer status and surveillance behaviors of mutation carriers and their relatives, and their needs for psychosocial, patient-provider, and family communication support. This is especially important over time, as little is known about decisional regret associated with genetic testing, communication, and support after the pathogenic variant has been identified in some family members but not in others, as well as impact on quality of life. A better understanding is needed of the overall response of the Swiss health care system to mutation carriers’ needs for long-term coordination of cancer surveillance and prevention. Finally, there are no interventions culturally tailored for Swiss families and designed to enhance patient-provider and patient-family communication, coping, and provide decisional support.

Establishing a registry with families harboring germline pathogenic variants associated with HBOC and LS and the collection of cancer surveillance and psychosocial data over time will greatly assist in finding sustainable solutions and developing cutting-edge interventions that optimize the health care system. However, establishing cascade screening for HBOC and LS and promoting interventions for communicating hereditary cancer risks pose several challenges at the medical and social level, requiring interprofessional collaboration with stakeholders from basic research, the health care system, and social science. In response to this challenge, the Swiss Cancer Genetic Predisposition Cascade Screening Consortium was assembled in 2015 with stakeholders from various disciplines (ie, basic science, epidemiology, medicine, nursing, psychology, public health, and sociology) to conduct the CASCADE study and examine the feasibility of establishing a family-based registry and a cohort with HBOC and LS mutation-harboring families.

The specific aims of the CASCADE study are to (1) survey index cases with HBOC or LS from clinic-based genetic testing records and determine their current cancer status and surveillance practices, needs for coordination of medical care, psychosocial needs, patient-provider and patient-family communication, quality of life, and willingness to serve as advocates for cancer genetic services to blood relatives, (2) survey blood relatives identified from pedigrees or family history records of HBOC and LS index cases and determine their current cancer and mutation status, cancer surveillance practices, needs for coordination of medical care, barriers and facilitators to using cancer genetic services, psychosocial needs, patient-provider and patient-family communication, quality of life, and willingness to participate in a study designed to increase use of cancer genetic services, and (3) explore the influence of patient-provider communication about genetic cancer risk on patient-family communication and the acceptability of a family-based communication, coping, and decision support intervention with focus group(s) of mutation carriers and relatives.

## Methods

### Design

CASCADE is a longitudinal study using surveys and focus groups, designed to elicit factors that enhance cascade genetic testing for HBOC and LS in Switzerland. The CASCADE study will contact known mutation carriers for HBOC and LS and systematically identify and contact their relatives to determine if they have had genetic testing, if they also carry the pathogenic variant, and how they manage their risk for hereditary cancer. Repeated observations are the optimal way for assessing these outcomes. The study will also use focus groups to examine the acceptability of a family communication, coping, and decision support intervention (Phase I). Table 1 presents a detailed description of assessments conducted for the study. The study protocol has been approved by the local ethics committee, while approval from ethics committees in other cantons is underway. The study will be carried out according to principles described in the Declaration of Helsinki and applicable Swiss laws and Swiss regulatory authority requirements.

### Setting

This multicenter study involves contributions from oncology and genetic testing centers from three linguistic regions of Switzerland (German-, French-, and Italian-speaking). Medical directors of clinical sites are either co-principal investigators (co-PIs) or site co-investigators and will oversee recruitment procedures according to the study protocol. The PI will oversee the scientific integrity of the study, including recruitment, data collection, and data analyses. These findings will be compiled and communicated to clinical sites.

### Sample and Sample Size

The CASCADE study targets individuals who have been identified through genetic testing as carrying a pathogenic germline variant associated either with HBOC or LS and their relatives (first- and second-degree, and first cousins). Textbox 1 describes applicable inclusion and exclusion criteria. Index cases include male and female cancer patients and cancer-free individuals. Cancer risk associated with HBOC and LS does not apply to children, thus, the study will include only adults (≥18 years old). Decisions to undergo genetic testing for these conditions are made by adults deemed competent to provide informed consent and should be undertaken after individuals participate in consultation regarding the benefits and drawbacks of genetic testing. Vulnerable participants (eg, those living in nursing homes) will be excluded because they may not be able to consent to genetic testing or follow recommended cancer surveillance or preventive measures. Critically ill patients will be excluded from recruiting relatives and from focus groups to avoid increasing subject burden.

**Table 1:** Flow of assessments for the CASCADE study.

Phase and steps		Tasks/Procedures	Data
**Selection of eligible index cases**			
	Random selection of families	Each clinical site provides the principal investigator (PI) with a list of the family identifications (IDs) determined by the clinical site as harboring a pathogenic germline variant. The PI randomly selects 35% of family IDs from the list with computer-generated numbers. The number of selected family IDs at each site is based on total number of family IDs at the clinical site and stratification for representative sampling.	No identifiable data for index cases are shared with the PI.
	Identification of eligible index cases	Through pedigrees and family history records, each site coordinator identifies index cases (1st family member to be identified as a carrier of a germline pathogenic variant) and determines whether they can be contacted (ie, alive and living in Switzerland). If an index case cannot be contacted, site coordinators identify 1st degree relatives who carry the familial pathogenic variant, randomly select one of them (computer-generated numbers), and determine whether they can be contacted. The process is repeated until an eligible mutation carrier is identified that can initiate cascade screening in the family.	Clinical sites collect minimal data (except identifiable data) for all index cases, regardless of whether they can be reached or not. Minimum data include gender, age, mutation, cancer type, age at diagnosis, stage, age tested, alive, place of residence, preferred language.
**Recruitment of eligible index cases**			
	Recruitment package to index cases	The medical director of each clinical site (co-PI or site co-investigator) and the site coordinator mail recruitment packages to index cases. If the index case did not receive genetic counseling at the testing site, then the recruitment package is sent to the referring physician who is asked to pass it on. Three attempts will be made to contact index cases. The medical director will inform treating oncologists about the participation of index cases.	Unique identification coding scheme enabling identifying index cases, site they were recruited from, and type of hereditary cancer syndrome (HBOC or LS). Dates recruitment packages were sent to physicians, dates the response from Index cases was received, and recruitment attempts made.
	Engagement of index cases in the CASCADE study	The site coordinator receives the informed consent or participation refusal form from index cases. Index cases accepting participation receive the CASCADE survey in their preferred language and format (paper/pencil or online) from the PI. The PI creates a coding key for identifying participants and the Clinical Trials Unit creates a coding key for variables assessed in the CASCADE survey.	Identifiable information for index cases accepting participation is passed on from site coordinators to the PI. Response rate from index cases, acceptance to participate in various stages of the CASCADE study, reasons for nonparticipation and preferred language and format for survey.
	Survey from index cases	The PI and the data management team receive the completed survey from index cases either in paper/pencil or online.	Assessment of data quality in each format (eg, percent missing data, outliers). Assessment of instrument reliability (Cronbach alpha and principal component analysis). Number of eligible relatives. Number of eligible relatives the index case is willing to invite. Characteristics of relatives reported by the index case. CASCADE study outcomes.
**Recruitment of eligible blood relatives**			
	Identification of eligible relatives	Based on index cases’ response to the CASCADE survey, the PI identifies eligible blood relatives the index case is willing to invite. Information about relatives is cross-referenced with pedigrees and family history information from clinical sites.	Number of relatives and degree of relationship to the index case (1st or 2nd degree relative, or 1st cousin).
	Recruitment package to eligible relatives	The PI prepares recruitment packages for relatives and a personalized letter for each index case, explaining the recruitment process and asking them to pass on recruitment packages to relatives.	Unique identification coding scheme enabling matching members of the same family.
	Engagement of eligible relatives in the CASCADE study	The PI receives informed consents or participation refusal forms from relatives. Relatives accepting participation receive the CASCADE survey in their preferred language and format (paper/pencil or online).	Response rate from relatives, acceptance to participate in various arms of the CASCADE study, reasons for nonparticipation and preferred language and format for survey completion.
	Survey from relatives	The PI and the data manager receive the completed survey from relatives either in paper/pencil or online form.	Assessment of data quality in each format (eg, percent missing data, outliers). Assessment of instrument reliability (Cronbach alpha and principal component analysis). Number of eligible relatives willing to invite. CASCADE study outcomes.
**Focus groups**			
	Selection of index cases and relatives	A purposeful sample of index cases and relatives accepting participation in focus groups will be selected by the qualitative methodologist and the PI.	Characteristics of index cases invited in the focus groups (preferred language, gender, mutation, type of cancer). Characteristics of families invited in the focus groups (level of support and communication).
	Invitation letters for focus groups	The PI in collaboration with the qualitative methodologist will send invitation letters initially to index cases and then to families selected for the focus groups.	Acceptance rate.
	Focus groups	Focus groups are organized and completed under the auspices of the qualitative methodologist.	Narrative data from focus groups.

**Characteristics of the target populations.**

Inclusion criteria

- Living carriers of germline pathogenic variants associated with HBOC and LS, and their relatives (1st and 2nd degree, and 1st cousins)
- Have at least one living blood relative
- Both genders
- Age ≥18 years old
- Mentally/physically able to provide informed consent
- Cancer patients and cancer-free individuals
- Can read/speak German or French or Italian or English
- Currently living in Switzerland

Exclusion criteria

- Carriers of unclassified genetic variants in *BRCA1, BRCA2* or *MLH1, MSH2, MSH6, PMS2, EPCAM* genes
- Currently not living in Switzerland
- Critically ill patients not able to complete the survey
- Not able to provide an informed consent
- Institutionalized (eg, nursing homes) or incarcerated

**Figure 1:**
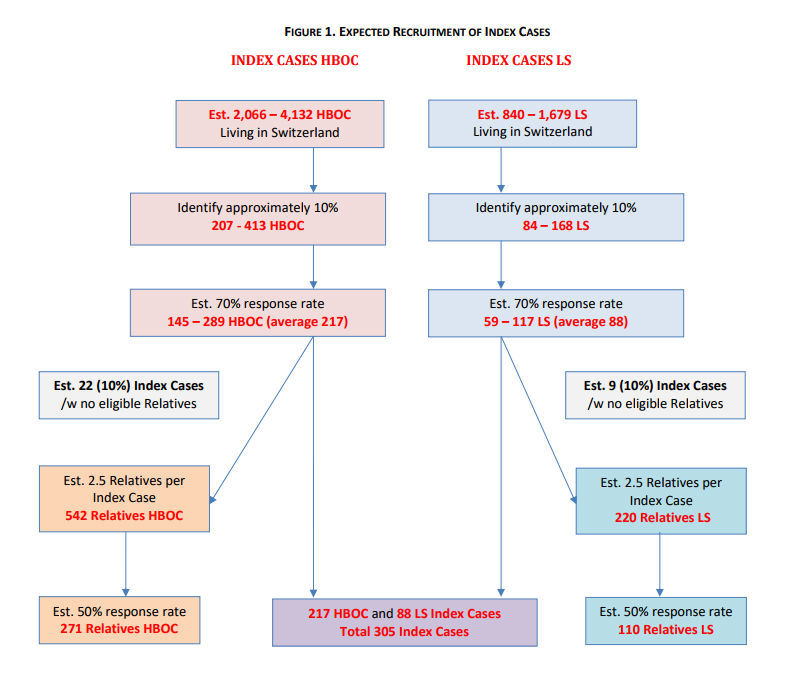
Expected recruitment of index cases.

Estimates of sample size are based on the PI’s experience, consultations with medical directors of clinical sites, and assuming average prevalence rates of 5% for hereditary breast cancer and 2-5% for hereditary colorectal cancer (the two most common manifestations of HBOC and LS), respectively. Assuming that is feasible to recruit 10% of mutation carriers from each participating clinic, 300 index cases will be targeted for inclusion within 12 months. It is expected that around 70% of approached index cases will accept participation, meaning that 495 index cases need to be approached to reach 305. It is estimated that from each index case we will identify 2.5 relatives. Assuming a response rate of 50% among relatives, we expect to recruit approximately 381 relatives. Figure 1 presents the CONSORT [68] diagram for recruitment of expected index cases and relatives.

### Recruitment Procedures for Index Cases

Participating Swiss clinical sites will record the total number of mutation-harboring families for HBOC or LS. A dedicated staff person at each clinical site, the site coordinator, will identify eligible index cases (ie, first person in the family identified as carrying a germline pathogenic variant associated with HBOC or LS), determine whether they can be contacted or not, and initiate and monitor the recruitment process. Selection of mutation-harboring families from each clinical site will involve the following steps:

1. Each site creates a list with IDs (eg, 001…, 350) corresponding to each index case and a family with a pathogenic variant.
2. The ID list is sent to the PI, who selects approximately 35% of cases with the assistance of a computer-generated random list. Identifiable information is not released until the index case accepts participation through signing an informed consent form.
3. Site coordinators retrieve the medical charts of selected index cases and decide whether the cases can be contacted by determining living status and residence.
4. If the index case is not available, then site coordinators identify first-degree relatives, who have also been identified through genetic testing as carriers of the familial pathogenic variant, and randomly (computer-generated list) select one of them.
5. Steps 3 and 4 are repeated until an index case who can be the initial contact person for the family is identified. If this process yields no results, the next family is selected.
6. All information obtained from each step is recorded, including minimum information for index cases. Minimum information includes demographics (age, gender), clinical history (tumor type, age at diagnosis, stage), and genetic testing results (including MSI and IHC tumor testing for LS patients) and is obtained from medical records. Index cases are recruited to the CASCADE study by the medical director of the respective site.

Index cases will be mailed an information letter, two copies of the informed consent form, two copies of a participation refusal form, and a stamped self-addressed envelope to return their response to the clinical site. Index cases will be informed about the objectives of the CASCADE study, participation requirements, the study plan, confidentiality, and associated risk and benefits through the informed consent form, which explicitly requires their agreement to (1) complete the CASCADE survey, (2) contact one or more of their blood relatives for the study, (3) be contacted once a year for 5 years and provide updated information about their health, and (4) participate alone or with a blood relative in a focus group. Index cases can participate in all or in some of the above study steps. The information letter explains that the minimum requirement for taking part in the CASCADE study is to complete the self-administered survey once. The refusal form asks nonparticipating index cases the reason for their refusal; this information is necessary for the validity of the study.

Site coordinators will determine whether the identified index case can be contacted or not by investigating whether they are alive and whether they live in Switzerland through hospital and civil records. If the recruitment package is returned undelivered, additional address verification methods will be used to locate a new residence. If the index case cannot be contacted a priori, coordinators will determine whether a first-degree relative can be the new index case for the family. Three attempts will be made to contact index cases for each family. If the study receives no response 6 weeks after the third attempt, a new family will be selected to preserve required sample size. Index cases will be recruited to the CASCADE study on a consecutive ongoing basis. Site coordinators will review pedigrees and family history of index cases who accept participation to extract demographic and medical information and to record all blood relatives (first- and second-degree relatives and first cousins). Index cases will be asked to complete a self-administered survey.

When an index case has not received genetic counseling at the participating center, the invitation package for the CASCADE study will be sent by the referring physician. This is necessary because some clinical sites perform only genetic testing and the referring physician is considered the medical person who has direct knowledge of index case’s genetic testing results. Site coordinators and the PI will keep track of the recruitment process. Referring physicians will make three recruitment attempts by mailing a new invitation package every 6 weeks if the index case does not respond to the invitation (either positively or negatively). Contact information (address, telephone, email) of the PI and the medical director will be provided in the information letter, so that index cases can request further information about the study at any point. A signed informed consent will be requested prior to index case’s enrollment as a prerequisite for engagement in the CASCADE study.

### Recruitment Procedures for Relatives

In order to alleviate ethical concerns associated with contacting blood relatives (ie, first- and second-degree relatives, and first cousins) without their explicit consent, the CASCADE study will approach them through index cases and will approach only relatives the index case is willing to contact. This recruitment method has been used in previous family-based studies with very good recruitment outcomes [69,70]. Index cases will be mailed recruitment packages to pass on to their relatives, including an information letter, two copies of the informed consent form, two copies of the participation refusal form, and a stamped self-addressed envelope for relatives to return their response to the PI. Relatives’ identifiable information will not be released to the PI. By returning a signed informed consent, the relative indicates willingness to participate and releases their identifiable information to the PI. Once this information is available, a recruiter will contact them to ascertain eligibility. If relatives do not respond after 6 weeks, the PI will contact the index case asking them to pass on a reminder letter to the nonresponding relative. If this effort yields no response, there will be no further attempts to contact the relative. Relatives agreeing to participate will receive a similar survey as the index case, asking if they are willing to (1) invite additional relatives to the CASCADE study, (2) be contacted once a year for 5 years and provide updated information about their health, and (3) participate alone or with a blood relative in a focus group. Relatives can also participate in all or some of the above study steps.

### Recruitment Procedures for Focus Groups

Two series of focus groups will be organized to explore the (1) difficulties associated with patient-provider communication regarding genetic cancer risk, (2) difficulties associated with patient-family communication regarding the pathogenic mutation, (3) mutual influence of patient-provider and patient-family communication, and (4) acceptability of a family-based intervention designed to enhance communication, coping, and decision making for genetic testing. A purposeful sample of index cases and relatives will be selected from individuals who agreed to participate in focus groups. The sampling method will be based on the expertise of the qualitative methodologist from interviews with Swiss *BRCA* carriers [32,33,71] and the PI’s experience conducting focus groups with US *BRCA* families. Segmentation strategy will guide sampling methods and the composition of the focus groups. Each focus group will be relatively homogeneous, while the full set will include several potentially distinct perspectives [72]. Focus groups will include 5-10 participants. Male and female cancer patients and cancer-free individuals will be selected to represent HBOC and LS.

It is expected that data saturation will be reached with 6-10 focus groups including about 30-60 carriers and 30-60 relatives. The first series of focus groups will include only mutation carriers stratified according to level of family communication (high, intermediate, low). These focus groups will explore the difficulties in patient-provider and patient-family communication, and the interrelatedness of these two types of communication. The second series of focus groups will include carriers and relatives and will explore the acceptability of an intervention designed to facilitate communication of test results among family members, helpful coping mechanisms, and decision making for genetic testing. Two sampling methods are envisioned. One method involves several members of the same family who can be invited together; the other involves 3-4 family pairs consisting of one carrier and one relative, which will be homogeneous in terms of gender, health status, etc. The sampling method of the second series of focus groups will be informed by responses to the CASCADE survey and findings from the first series of focus groups.

### Data Collection and Data Management

The CASCADE survey will be developed in English, translated into three languages (German, French, and Italian), and back-translated into English by professional translators, except for scales with validated versions in these languages (eg, 12-Item Short Form Health Survey [SF-12]). Discrepancies will be resolved by the PI with the collaboration of the translators and the co-investigators. Index cases and relatives will be given the choice to complete the CASCADE survey either as paper/pencil or using an online platform. The content of the paper/pencil and online survey are identical. Participants who choose to complete the survey online will receive an access code and will be instructed how to log into a secure Web platform. If a survey is missing important information (eg, number of relatives the index case is willing to contact), research personnel will contact participants to ascertain it.

No identifying information, such as name and address, is collected with paper/pencil or online surveys. Each index case is given a code; for example, G001-IC stands for an index case selected from the Geneva clinic with the family study code 001. Relatives recruited from this index case will be coded G001-R1, G001-R2, etc, to establish the link between family members. This code will be used for surveys, consent forms, refusal forms, and correspondence letters to match participants to the correct family unit and maintain the study’s internal validity. The PI and coordinators will keep logs with these codes. The coding key will be kept in a password-protected computer file and will be available to the PI, members of the Swiss Cancer Genetic Predisposition Cascade Screening Consortium, and key personnel. The code will be broken only to avert an immediate risk to the health of the person, in cases of withdrawal from the study, or when there is a legal basis.

All study data will be collected and stored in a secure database and handled by the data management team from the Clinical Trials Unit, University Hospital, Basel (CTU Basel). The online survey will be implemented using LimeSurvey, installed on a separate server, and exclusively used for the study. Lime Survey is an established app to perform online surveys. The system (server and data) is integrated in a regular backup process. Data transfer from and to the Web-based survey system are encrypted using secure sockets layer/transport layer security (SSL/TLS). The secure database will be used for data collection and to track returned surveys. Data entered for paper/pencil surveys will be double-checked for accuracy. The usability of the paper/pencil or online survey will be assessed based on number of individuals who choose either mode, percent of missing data, etc. Many items are parts of multi-item scales and are anticipated to correlate with each other. The reliability of these scales will be tested using principal component analyses and Cronbach alpha coefficients. Scales with alpha≥.71 will be used. On completion of approximately 30 surveys, scale psychometrics will be examined. For any given scale that shows less than required psychometric properties (ie, Cronbach alpha<.71 and factor analysis indicates item loadings <10% compared to item loadings in the original scale), a revision of the translated scale will be undertaken. This will allow comparisons of scale reliability based on delivery mode and will establish whether the survey can be administered interchangeably.

Health-related and personal data collected for the CASCADE study are confidential; coding will safeguard participants’ confidentiality. All study documents will be archived in the PI’s office. Site-related documents will be archived at the office of each medical director. Administrative data are accessible only by authorized personnel and data managers from CTU Basel. Direct access to documents will be permitted for monitoring, audits, or inspections. Ethics committee members, members of the Swiss Cancer Genetic Predisposition Cascade Screening Consortium, the statistician, and key personnel will have access to project plan, dataset, statistical code, etc, during and after the study (publication, dissemination). Paper/pencil surveys will be stored in a separate research office in the PI’s building for 5 years and then destroyed by shredding. Once all data have been collected, the complete dataset and survey setup will be exported by CTU Basel and transferred to the PI and the statistician via a secure channel. The survey system (including database) will be purged after the end of the study. The PI will archive the electronic data for a minimum of 10 years.

### Outcomes

Table 2 [73-89] describes primary outcomes for index cases and relatives and the scales used to assess them. The feasibility of establishing a family-based registry will be assessed using the number of mutation-harboring families associated with HBOC and LS from each clinical site, the number of relatives identified from pedigrees and family history, index cases’ response rate to the CASCADE survey, the number of relatives each index case is willing to invite, relatives’ response rate to the CASCADE survey, and the willingness of index cases and relatives to be contacted once a year for 5 years. Additional outcomes include assessing acceptance rates of paper/pencil and online platform and quality of data (eg, percent missing values).

**Table 2:** Scales used in the CASCADE survey.

Concepts		Scale	Index case	Relatives
Demographics		Age, gender, education, employment status (previously used) [73]	√	√
**Health history**				
	Comorbidities	Chronic conditions associated with mobility, cardiovascular disease, diabetes, anxiety, depression Self-reported list (yes/no) (previously used) [73]	√	√
	Reproductive history (females)	Risk factors associated with the Gail model [74,75] Self-reported	√	√
	Alcohol, tobacco, physical activity	Self-reported (previously used) [76]	√	√
**Cancer-related**				
	Cancer diagnoses	Type of cancer, age of onset Self-reported list (previously used) [73]	√	√
	Surgery	Surgeries associated with HBOC & LS Prophylactic surgeries Self-reported (previously used) [29]	√	√
	Surveillance behaviors	Surveillance for cancers associated with HBOC & LS Surveillance for common cancers Investigating tool developed per the American Society of Clinical Oncology guidelines [77] (previously used) [73]	√	√
		Barriers & facilitators (previously used) [73] Coordination of medical care (multiple choices) High out-of-pocket costs (yes/no)	√	√
	Family history	Family history in 1st and 2nd degree relatives & 1st cousins – type of cancer, age of onset (previously used) [73]	√	√
**Psychosocial needs**				
	Fear of cancer recurrence	Concerns About Recurrence Scale [78] 4 items, 7-point Likert scale	√	√
	Perceived cancer risk	Perceived Risk for Developing Cancer [79] 1 item, 10 points with verbal anchors	√	√
	Decisional conflict	Decisional Conflict associated with genetic testing [80] 16 items, 7-point Likert scale		√
	Decisional regret	Decisional Regret associated with genetic testing [81] 5 items, 7-point Likert scale	√	
	Coping with stressful events	Brief Cope [82] 25 items, 7-point Likert scale	√	√
	Self-efficacy	Self-efficacy dealing with cancer [83] 14 items, 7-point Likert scale	√	√
		Self-efficacy – use genetic services (counseling & testing) [83] 1 item, 7-point Likert scale		√
	Knowledge	Breast & Ovarian Cancer Risk Factor Knowledge Index [84,85] 17 items (True, False, Don’t Know)	√	√
		Knowledge of Breast Cancer Genetics Scale [70] 12 items (True, False, Don’t Know)	√	√
		LS Risk Factors & Inheritance Investigator developed 19 items (True, False, Don’t Know)	√	√


**Communication**				
	Physician	Need for physician communication about mutation Investigator developed 10 items, 7-point Likert scale	√	
	Family	Mutuality & Interpersonal Sensitivity [86] 15 items, 7-point Likert scale	√	√
		Family Support in Illness [73,87] 10 items, 7-point Likert scale	√	√
		Communication with children & relatives about mutation (previously used) [29] 17 items (multiple choice)	√	
**Genetic services**				
	Genetic services	Barriers & facilitators (previously used) [29,88] 11 items, 7-point Likert scale & 22 items (multiple choice)	√	√
	Genetic testing	Had genetic testing (yes/no) Self-reported	√	√
	Referral	Source & involvement (previously used) [29] 16 items (multiple choice)	√	
	Quality of Life	SF-12 [89] Physical component & Mental component	√	√

### Data Analyses

Selection bias will be minimized by random selection of mutation-harboring families in each clinical site from three linguistic regions of Switzerland. Stratification will ensure selection of an equal proportion of index cases from clinical sites that offer genetic services for both syndromes. The study will try to recruit all index cases from clinical sites including fewer than 100 mutation-harboring HBOC/LS families to ensure a representative sample.

All statistical analyses will be conducted in licensed software packages, including Microsoft Excel, SPSS (IBM), and R. For all statistical tests, significance will be set at two-sided alpha=.05. Data values will be examined for legality (within appropriate range) using histograms and box plots and corrected when possible. Descriptive analyses will include calculating means, standard deviations, frequencies, and percentages of variables and participant descriptors. Bivariate analyses (Pearson correlations, chi-square test for differences in proportions, and *t* test for differences in means) will assess associations between demographics and clinical characteristics. Regression analyses will incorporate generalized estimating equations for pairing index cases with their relatives and explore to what extent predictors are in direct, mediating, or moderating relationship to an outcome.

The following comparisons will take place: between index cases and relatives, between HBOC and LS, between men and women, cancer patients versus cancer-free individuals, participants with children versus those with no children, between different age groups and different cancer diagnoses. Data from participants who withdraw will be kept in the study to ensure the internal validity of the study. Missing data from multi-item scales will be addressed with multiple imputations using R software if they exceed 5% of observations and if they are less than 25% for each specific scale. Scale reliability will be assessed with Cronbach alpha and principle component analyses. Deviations from the planned analyses are not foreseen. The study statistician will review and approve any deviations from the original statistical plan if necessary.

Narrative data from focus groups will be recorded and transcribed verbatim to allow data management and content examination. Thematic analyses to inductively classify data in concepts and categories, as these emerge through an interpretive process, will be carried out under the guidance of the qualitative methodologist [90]. Focus group participants will be shown a prototype of a family-based intervention as a PowerPoint presentation. Then they will be asked if they like the intervention, if they find it useful, and how it can be improved. Acceptability of the intervention will be assessed with a short survey using 7-point Likert-type items (1=Low to 7=High) asking overall satisfaction with the content, format and appearance of the program, and whether it can help with family communication, coping, and decision making. The survey assesses six acceptability items: ease of use, clarity, appropriate length, appropriate level of detail, able to hold interest, and satisfaction.

## Results

This study is currently recruiting participants.

## Discussion

### Principal Considerations

Cancer predisposition cascade genetic screening combines personalized medicine and public health. Once a mutation carrier for HBOC or Lynch syndrome is identified, evidence-based interventions are available that can reduce the risk of adverse health outcomes in entire cohorts of relatives [91]. This approach is cost-effective for Tier 1 genetic conditions, leading to reduced medical and insurance coverage costs (eg, treatment and hospitalization expenses) [92-94]. Cascade screening for FH applied in the Netherlands identified thousands of mutation carriers for the disorder and has been subsidized by the Dutch government since 2015 [95,96]. Similar programs for FH have also been implemented in Scotland and Wales [97,98].

Availability of genetic testing created an increasing demand for coordination of health care services and risk communication among index cases and relatives. Knowledge of hereditary risk can serve as an information tool to reduce cancer morbidity and mortality. This necessitates the establishment of family-based registries that systematically record genetic information. Currently, this information is fragmented and dispersed across Swiss clinical sites. The establishment of high-risk clinics would allow synergistic approaches in cancer surveillance and medical care offered to these families. Effective data sharing and dissemination across disciplines is mandatory for increasing the impact of genetic screening, ensure resource allocation, and facilitate health care policy and decision making.

### Conclusion

CASCADE study will promote multidisciplinary research in public health genetics at the cutting edge of medicine with strong translational application. This has significant potential to enhance the development of high-quality comprehensive support systems to improve use of cancer genetic services and facilitate patient involvement in health care decisions. The long-term outcome of this program is the development and implementation of new models for systematic surveillance and detection of individuals at risk for hereditary cancer in Switzerland. Immediate outcomes are the assessment of current use of cancer genetic services and evaluation of the public health impact of HBOC and LS. The CASCADE study will document the needs of mutation-harboring families, including barriers and facilitators to accessing cancer genetic services, and will promote use of family history for genetic risk assessment. The study will also provide information for the acceptability of an intervention that will potentially increase genetic literacy, expand understanding of health care technologies, and reduce HBOC- and LS-related morbidity and mortality in Switzerland.

## Abbreviations

BRCA1: breast cancer 1
BRCA2: breast cancer 2
CTU: Clinical Trial Unit
FH: familial hypercholesterolemia
HBOC: hereditary breast/ovarian cancer syndrome
HFTA: Federal Act on Human Genetic Testing
LS: Lynch syndrome
MMR: mismatch repair
MSI-H: microsatellite instability
